# Survival after prolonged resuscitation with 99 defibrillations due to Torsade De Pointes cardiac electrical storm: a case report

**DOI:** 10.1186/1757-7241-18-7

**Published:** 2010-02-06

**Authors:** Anders Rostrup Nakstad, Christian Eek, Dag Aarhus, Anne Larsen, Kristina Hermann Haugaa

**Affiliations:** 1 Department of Anesthesia and Air Ambulance Department, Oslo University Hospital - Ullevål, Oslo, Norway; 2 Department of Cardiology, Oslo University Hospital - Rikshospitalet and University of Oslo, Oslo, Norway; 3 Department of Anaesthesia, Vestre Viken HF - Drammen, Norway; 4 Department of Internal Medicine, Vestre Viken HF - Drammen, Norway

## Abstract

A 48-year-old previously healthy woman suffered witnessed cardiac arrest in hospital. She achieved return of spontaneous circulation and was transferred to the intensive care unit. During the following 3 hours, she suffered a cardiac electrical storm with 98 episodes of Torsade de Pointes ventricular tachycardia rapidly degenerating to ventricular fibrillation. She was converted with a total of 99 defibrillations. There was no response to the use of any recommended anti arrhythmic drugs. However, the use of bretylium surprisingly stabilized her heart rhythm and facilitated placing of a temporary pacemaker. Overdrive pacing prevented further arrhythmias and was life saving. A number of beneficial factors may have contributed to the good neurological outcome. Further investigations gave no explanation for her cardiac electrical storm.

## Background

Torsade de Pointes (TdP) cardiac electrical storm may be defined as the occurrence of more than two distinct episodes of destabilizing TdP in 24 hours [1,2]. It is a rare but challenging medical emergency and effective treatment may be difficult, especially when TdP degenerates to VF or when the arrhythmia is a symptom of underlying cardiac disease [3]. TdP ventricular tachycardia was first described in 1966 [4]. A number of causes are associated with TdP, including inherited long QT-syndrome, female gender and some acquired conditions like use of anti-arrhythmic drugs (especially class Ia and III), electrolyte disturbances, heart failure, subarachnoidal haemorrhage and hypothermia [2,5]. Magnesium sulfate is recommended as the first line of therapy, in addition to beta-blocker therapy [6-8]. In this report we describe a case were a previously healthy woman suffered a dramatic period of multiple events with TdP degenerating to VF.

## Case presentation

A 48-year-old woman was admitted to the neurological department due to sudden loss of consciousness and seizures from which she had recovered spontaneously. The primary survey revealed no cardiac or neurological abnormalities, and she was tentatively diagnosed to have suffered from an epileptic seizure. She reported to have experienced a similar incident one month prior to admission. This self-limiting seizure was witnessed by a relative. She had no other history of disease or discomfort of any kind.

**Figure 1:**
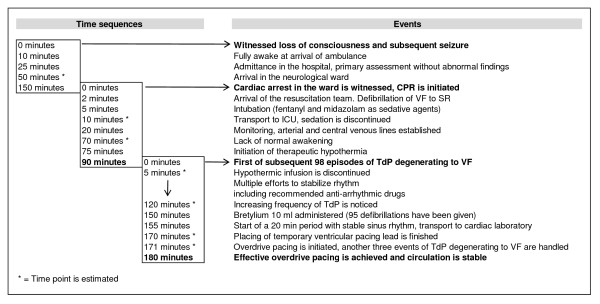
**The time-line of events, based on the available documentation**.

Figure 1 illustrates the time-line of events. Approximately two hours after admittance she was found pulseless in the ward. Basic cardiopulmonary resuscitation (CPR) was immediately started. A resuscitation team arrived after approximately 2 minutes and successfully defibrillated a VF into sinus rhythm. Return of spontaneous circulation (ROSC) was confirmed but the patient did not regain consciousness. She was intubated and transferred to the Intensive Care Unit (ICU) where therapeutic hypothermia was initiated, in compliance with current recommendations [9]. Arterial line, central venous line, twelve-lead ECG, controlled mechanical ventilation and capnography were established. She was sedated with midazolam and fentanyl. Cooling was initiated by use of external cold blankets and infusion with Ringer's solution (4°C) at a rate of 100 ml per minute via a peripheral venous line [10].

Ninety minutes after her first cardiac arrest in the ward - with an oesophageal temperature of 36.8°C - she suffered a new event of ventricular arrhythmia. Immediate chest compressions were started and charging of the defibrillator initiated. Manual chest compressions maintained a systolic blood pressure of above 90 mmHg and adequate signal quality on the peripheral pulse oximeter was noticed. Adhesive pads were placed in standard positions (apex and upper right chest). A defibrillator with biphasic delivery of 150 Joule was used and successful defibrillation into sinus rhythm achieved on the first attempt.

**Figure 2:**
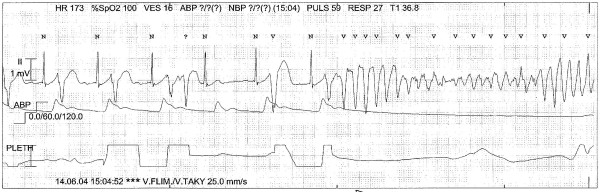
**The first documented episode of sinus rhythm (with multiple ventricular premature beats) spontaneously converting to Torsade de Pointes ventricular tachycardia initiating a sequence of chest compressions and (later) defibrillation**.

During the next 150 minutes the patient suffered from repetitive episodes of ventricular arrhythmia with an initial pattern of TdP that degenerated into VF. This pattern was typical for all subsequent arrhythmic events (Figure 2). Each event triggered immediate chest compressions for 20-30 s while charging the defibrillator. Each shock successfully converted her to sinus rhythm in all but one case (where the second shock was successful) with rapid normalization of invasive blood pressure values. The defibrillator was connected to a 220V DC outlet during the entire resuscitation to ensure its function. The adhesive pads were replaced after approximately 55 defibrillations to maintain connectivity.

**Figure 3:**
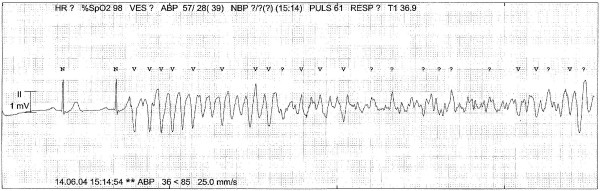
**Typical record with Torsade de Pointes ventricular tachycardia that rapidly degenerates into ventricular fibrillation**.

The arrhythmic events relapsed in cycles of approximately 20-240 seconds. Numerous ventricular premature beats were observed in the periods of spontaneous circulation. Because the TdP very rapidly degenerated to VF (Figure 3), the arrhythmia was first misinterpreted as simple VF. Thus the patient was intravenously administered amiodarone 300 mg twice without effect on event frequency. When the diagnosis of TdP was made, administration of lidocain 100 mg, metoprolol 15 mg and repeated doses of magnesium sulphate was tried. Despite no sign of myocardial infarction on ECG reteplase was also administered after an initial dose of heparin. None of these efforts reduced the frequency of recurring arrhythmias, but rather decreased duration of sinus rhythm.

After 150 minutes in the ICU, a total of 95 defibrillations had been given. The desperate situation motivated the infusion of 10 ml of bretylium (Bretylate^®^, bretylium tosylate 50 mg/mL, GlaxoSmithKline) that was provided from the department of anaesthesia. Surprisingly, after few minutes the occurrence of TdP arrhythmia terminated. During the subsequent 20-minute period the cardiologists were able to insert a temporary right ventricular pacing lead. TdP reoccurred 3 times after the procedure but ceased after overdrive pacing was set to a frequency of 130 beats/min. At this time the patient had been defibrillated 99 times, and approximately 180 minutes had elapsed since her cardiac arrest in the ICU.

The patient was transferred to a tertiary centre where a coronary angiography showed normal coronary arteries. Chest x-ray was normal and there was only a slight increase in cardiac enzymes (Troponin I: 0.22 ng/mL). Therapeutic hypothermia was again introduced and sustained for 24 hours, without any recurrent arrhythmia. After two days, sedation was discontinued and she regained consciousness with intact cerebral function. An implantable cardioverter defibrillator (ICD) was implanted two weeks later.

The complete set of cardiovascular investigations including molecular genetic analyses did not reveal any explanation for the patient's TdP cardiac electrical storm. She had not used any drugs known to prolong QT interval prior to admission. Five years after the described event no ventricular arrhythmias have been detected by the ICD. She has continued long-term treatment with metoprolol succinate and has returned to fulltime work.

## Discussion

This case demonstrates that seemingly desperate long term resuscitation may sometimes be successful. We found the arrhythmia free interval of 20 minutes shortly after administration of bretylium remarkable and crucial for the subsequent insertion of a pacing lead. Bretylium is a class 3 anti-arrhythmic drug that was excluded from ERC Guidelines of resuscitation due to lack of sufficient evidence. Case reports are conflicting [11] while there are reports of effectiveness of bretylium in treatment of sustained ventricular arrhythmias [12,13]. Multiple drugs were given in the 150 minutes prior the arrhythmia free interval, thus it may of course be argued that the stable period was independent of the use of the bretylium.

The use of overdrive pacing to suppress arrhythmias in patients suffering from reoccurring or sustained TdP was suggested more than three decades ago and is confirmed in recent literature [14,15]. We believe it is important to acknowledge the difference between patients with spontaneous circulation (where ordinary ECG-sampling is possible) and patients where TdP very rapidly degenerates into VF. In our patient the rapid degeneration into VF delayed the precise diagnosis of the arrhythmia. Sophisticated monitoring in the ICU made it possible to identify the TdP and thus made overdrive pacing strongly indicated. However, despite the precise diagnosis, frequent periods of chest compressions and defibrillations made the intervention difficult to perform. During the remarkable 20-minute period of sinus rhythm the cardiologists were able to insert a temporary right ventricular pacing lead.

The patient reported to have experienced a similar incident of sudden loss of consciousness and seizures one month prior to admission. Self-terminating episodes of TdP are reported to cause hypotension and seizures, probably due to cerebral hypoperfusion [16] and may be suggested as a reason for the incident that made the patient admitted to hospital.

The increased frequency of TdP observed may have been triggered by the combination of anti-arrhythmic drugs that were given in the attempt to stabilize heart rhythm. Several anti arrhythmic drugs, including amiodarone, have pro-arrhythmic effects [14,17]. In addition, it may be speculated if the myocardial hypoxia suffered during the primary cardiac arrest and the initiation of therapeutic hypothermia may have contributed to the TdP cardiac electrical storm. The latter is not likely, because the oesophageal temperature was almost normal (36.8°C) at the time.

The good neurological outcome in the patient was probably due to a number of positive factors. This case illustrates that immediate and high quality CPR can sustain a subnormal systolic BP of 90 mmHg observed by the calibrated intra-arterial pressure wave curve. Each episode of VF initiated immediate manual chest compressions while charging the defibrillator, thus hands-off time was reduced to a minimum. Use of end-tidal CO_2_-monitoring made it possible to secure a normal frequency and tidal volume of ventilation in periods of normal circulation. Adequate end-tidal CO_2_-values detected during chest compressions motivated the prolonged efforts. The intervals between recurrences of TdP were between 20 to 240 seconds. Thus the patient had frequent periods of beneficial spontaneous circulation during the resuscitation period.

## Conclusions

Various recommended anti-arrhythmic drugs did not terminate the TdP cardiac electrical storm in our patient. The use of bretylium may have facilitated an arrhythmia free interval and may be considered as a supplementary drug when recommended medication has been insufficient. Overdrive right ventricular pacing prevented new arrhythmic events and was life saving. A combination of unknown predisposing factors, hypoxia and use of multiple drugs may have acted pro-arrhythmic. This case may serve as a reminder that a good neurological outcome is possible despite prolonged resuscitation efforts.

## Abbreviations

TdP: Torsade De Pointes; VF: Ventricular Fibrillation; CPR: Cardiopulmonary Resuscitation; ROSC: Return of Spontaneous Circulation; ICU: Intensive Care Unit; BP: Blood Pressure; CO_2_: Carbon dioxide.

## Consent

Written informed consent was obtained from the patient for publication of this case report. A copy of the written consent is available for review by the Editor-in-Chief.

## Competing interests

The authors declare that they have no competing interests.

## Authors' contributions

ARN, DA, CE, KHH and AL were all involved in treating the patient and gathering of clinical data. All authors made substantial contributions in drafting the manuscript, and have given final approval of the final version to be published.
